# A Biocompatible Near-Infrared 3D Tracking System*

**DOI:** 10.1109/TBME.2017.2656803

**Published:** 2017-01-23

**Authors:** Ryan S. Decker, Azad Shademan, Justin D. Opfermann, Simon Leonard, Peter C. W. Kim, Axel Krieger

**Affiliations:** Children's National Health System, Washington, DC 20010 USA; Children's National Health System, Washington, DC 20010 USA; Children's National Health System, Washington, DC 20010 USA; Computer Science Department, Johns Hopkins University, Baltimore, MD 21218 USA; Children's National Health System, Washington, DC 20010 USA; Children's National Health System, Washington, DC 20010 USA

## Abstract

A fundamental challenge in soft-tissue surgery is that target tissue moves and deforms, becomes occluded by blood or other tissue, and is difficult to differentiate from surrounding tissue. We developed small biocompatible near-infrared fluorescent (NIRF) markers with a novel fused plenoptic and NIR camera tracking system, enabling 3D tracking of tools and target tissue while overcoming blood and tissue occlusion in the uncontrolled, rapidly changing surgical environment. In this work, we present the tracking system and marker design and compare tracking accuracies to standard optical tracking methods using robotic experiments. At speeds of 1 mm/s, we observe tracking accuracies of 1.61 mm, degrading only to 1.71 mm when the markers are covered in blood and tissue.

## I. Introduction

Accurate and precise tracking of tools, tissues, and dynamic background scenes is increasingly important in computer-assisted surgery, providing intraoperative guidance, motion stabilization, and dynamic constraints for increased safety and better surgical outcome [1], [2]. In contrast to rigid tissue surgery such as surgeries on bone, soft-tissue procedures remain elusive to robust tracking methods. In addition, respiratory and cardiac motion further hinder dexterous tasks such as vessel anastomosis, and must be accommodated by the surgeon or robotic platform [3]. Methods such as structured light or stereo approaches to track features on changing tissue surface geometry in real-time are hampered by interference, depth resolution and the homogeneous scenes present during surgery [4]–[7]. Robust and real-time tracking often relies on easily-detectable fiducials.

Optical trackers measure the coordinates of one or more special markers in the visual and near-infrared spectrum [8], [9]. One such tracker, commonly used in medical procedures, is the Polaris system (NDI, Waterloo, Canada). 3D marker position can be calculated by triangulation from two disparate views in a stereoscopic arrangement. Optical tracking can be very accurate and span large working volumes, but it requires a direct line of sight, and cannot calculate the position of an occluded marker.

There are other visual methods using high-contrast dyes or inks to differentiate tissue. For example, India ink can be tattooed to target tissues for optical tracking [10], but also suffers from occlusion limitations. Methods tracking visual features in color (RGB) images can have difficulty due to low correspondence between images, and features that are not robust [11]; they are also susceptible to occlusion problems.

To overcome the problem of occlusion, electromagnetic (EM) trackers [12], [13] generate a magnetic field and measure the induced voltages in a marker which contains oriented wire coils. The geometry of the coils is known beforehand, and can be used in conjunction with the induced voltages to calculate the position and orientation of the electromagnetic marker. Electromagnetic trackers do not require a line of sight to the target, but may be affected by metals or magnetic disturbances. They can create artifacts that interfere with MRI or CT imaging [12], and require the EM markers to be tethered to cables.

Gold markers [14] can be imaged by CT, MRI, and ultrasound, but don't show up in normal RGB surgical imaging. They are inert for use inside the body and often used to track cancerous tissue to help guide radiation therapy. Typically the markers are implanted directly in tissue; their use on top of soft tissue is limited because they do not adhere.

Near-infrared (NIR) imaging has the potential to overcome occlusion problems because NIR light penetrates deeper than visual light. NIR fluorescent (NIRF) imaging with Indocyanine green (ICG) has been shown to be very effective in detecting subsurface (<5 mm) liver tumors, intraoperative blood vessels and graft patency invisible to human eyes [15]–[20].

We developed small biocompatible NIRF markers that are easily attached to tissue in an irrigated surgical setting. Together with a novel fused plenoptic and NIR camera tracking system, the proposed NIRF markers enable 3D tracking of tools and target tissue while overcoming blood and tissue occlusion in the uncontrolled surgical environment. We have previously explored the feasibility of these markers in simple tasks such as visualization of markers occluded by tissue [21], and 2D placement of stitches in phantom tissue [22]. We have also separately explored 3D mapping and completion of simple surgical tasks using plenoptic cameras [23], [24]. More recently, we have used this tracking system to guide a smart tissue autonomous robot (STAR) in performing supervised autonomous robotic suturing of in-vivo porcine bowel, demonstrating improved burst pressures compared to standard surgeries [25].

In this paper, we present the NIRF marker design, improved hybrid NIR-3D tracking methods, and evaluate *tracking accuracy* against standard optical tracking methods using robotically-controlled reference trajectories. We investigate the performance of our fused NIRF-3D system while tracking at various speeds and occlusion conditions, and during clinically relevant ex-vivo and in-vivo situations. Tested speeds are informed by respiratory motion (Schweikard simulates the speed of respiratory motion between 3-10 mm/s [26]) and tool tracking applications (Ren observes laparoscopic tool motions to be 10 mm/s or less [27]).

## II. Materials and Methods

### A. Imaging System

**Figure 1:**
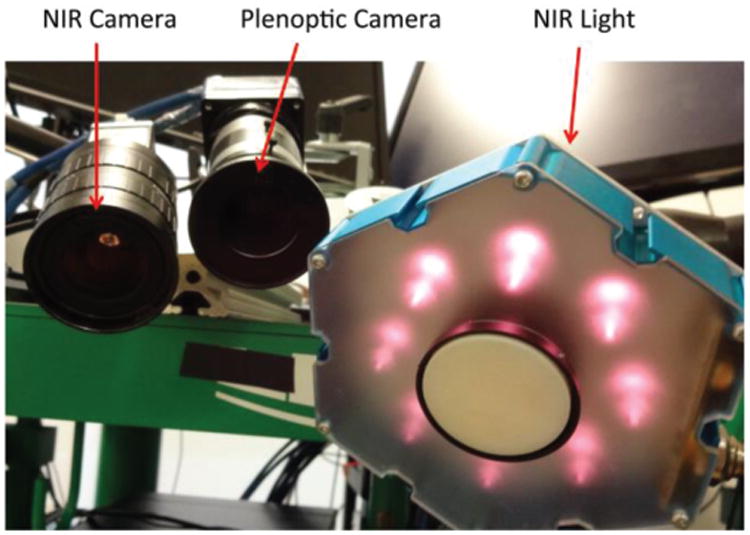
Camera and light arrangement, showing the Near-Infrared (NIR) camera used to track special Near-Infrared Fiducial (NIRF) markers in a 2D greyscale image. This camera is registered to the plenoptic camera, a 3D lightfield camera used to quantify the XYZ location of each marker in metric coordinates. The NIR light emits light at 760 nm to excite the special markers, making them glow brightly in the NIR image.

**Figure 2:**
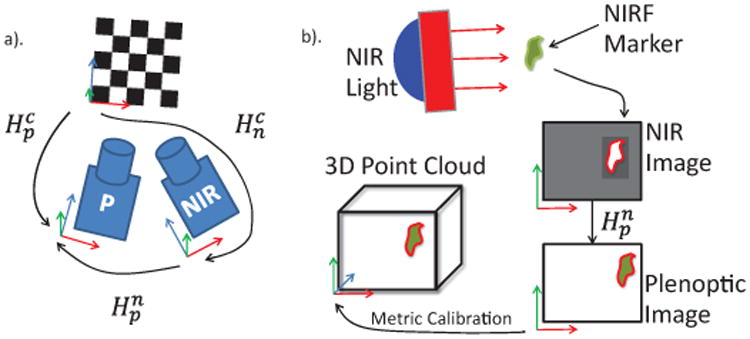
Schematic of NIR-3D system showing a) registration and b) NIR-3D tracking pipeline. Registration requires both cameras to observe a common checkerboard with known dimensions, from which the pose of each camera with respect to the checkerboard, and thus with respect to each other, is known. This allows points in one coordinate frame to be transformed into the other. The NIR-3D tracking pipeline is then constructed as follows: A marker is excited by the NIR light and observed in the NIR image. The marker coordinates are transformed into the plenoptic camera image. A metric calibration of the plenoptic camera translates the pixel coordinates to real world XYZ metric coordinates.

The imaging system consists of 2 cameras: an NIR-sensitive camera (acA-50gm 2000NIR, Basler Inc., Exton, PA) with CMOSIS CMV2000 2048×1088 pixel CMOS sensor running at 20 Hz, a plenoptic camera (R12, Raytrix GmbH, Schauenburgerstrasse, Germany) running at 5 Hz, and an NIR LED light source (760 nm, Marubeni America Corp, Santa Clara, CA) (Fig. 1). The NIR camera is equipped with a special 845 nm ± 55 nm bandpass filter (Chroma Technology Corp., Bellows Falls, VT) to pass excited NIRF light (Fig. 2). The plenoptic camera is a 3D light-field camera, which uses an array of microlenses to quantify the depth at each pixel; it has a factory calibrated field-of-view of 70×65×30 mm. The plenoptic camera has several advantages. First, it is a single lens depth imager, requiring less space than a traditional stereo tracker, important for use in the crowded operating room. Secondly, it allows the entire image to be brought into focus provided the scene is within the Field-of-View (FOV), something not possible with conventional single-lens cameras or stereo cameras, which is useful for tracking applications given the increased detection of fine local features.

### B. Camera Calibration

Camera calibration enables our vision system to translate measurements in pixels to real-world units such as millimeters. First, the intrinsic camera parameters such as focal length and principal point must be found, so that we may translate pixel coordinates to world coordinates in each camera coordinate frame. Secondly, extrinsic calibration (sometimes called registration) is required to find the position and orientation of the camera coordinate frames with respect to a common base frame, in our case a checkerboard pattern.

First, the intrinsic calibration of these cameras is done using Zhang's method [28], giving the intrinsic matrices *K* for the NIR (*n*) and plenoptic (*p*) cameras as seen in (1).

(1) $$K_{n,p}={\left[\begin{array}{ccc} f_{x} & 0 & C_{x}\\ 0 & f_{y} & C_{y}\\ 0 & 0 & 1 \end{array}\right]}_{n,p}$$

Where [*f_x_*, *f_y_*] are the *x* and *y* focal lengths, and [*C_x_*, *C_y_*] are the image coordinates of the principal point, where the optical axis intersects the image plane.

Secondly, the extrinsic parameters must be found for both cameras with respect to a common reference, in order to register the cameras to one another. This is accomplished with the camera pose package in the Robot Operating System (ROS) framework. A checkerboard (5 mm squares, 11×10 grid) is observed with both cameras, and the corner detection measurements are fed to a pose estimator, which publishes each camera's pose (*H_n_*, *H_P_*) relative to the checkerboard XYZ frame, where the pose is a 4×4 matrix combining a rotation and translation into the full homogeneous transformation between reference frames, shown in (2).

(2) $$H_{n,p}^{c}={\left[\begin{array}{cccc} R_{11} & R_{12} & R_{13} & t_{x}\\ R_{21} & R_{22} & R_{23} & t_{y}\\ R_{31} & R_{32} & R_{33} & t_{z}\\ 0 & 0 & 0 & 1 \end{array}\right]}_{n,p}$$

This can be broken into a 3×3 rotation matrix $R_{n,p}^{c}$ rotating the checkerboard XYZ axes to each of the camera XYZ axes, and a 3×1 translation vector $t_{n,p}^{c}$ from the checkerboard origin to each camera frame origin. The inverse rotation $R_{c}^{n,p}$ will align the camera frame axes with the checkerboard frame axes, and can be calculated as the matrix transpose, because rotation matrices are orthogonal. The inverse translation is $t_{c}^{n,p}=-t_{n,p}^{c}$

By knowing the pose of both cameras with respect to the checkerboard, the transformation between them $H_{p}^{n}=H_{p}^{c}H_{c}^{n}$ is also known (Fig 2a). For example, the transformation of a 3D point from the NIR to plenoptic camera frame is calculated as in (3).

(3) $${\left[\begin{array}{c} x\\ y\\ z\\ 1 \end{array}\right]}_{p}=H_{p}^{n}*{\left[\begin{array}{c} x\\ y\\ z\\ 1 \end{array}\right]}_{n}=R_{p}^{n}{\left[\begin{array}{c} x\\ y\\ z\\ 1 \end{array}\right]}_{n}+t_{p}^{n}$$

By a rotation $R_{p}^{n}$ and translation $t_{p}^{n}$, the transformation $H_{p}^{n}$ converts a 3D point location from the NIR camera frame to the plenoptic camera frame.

The plenoptic camera also requires a metric calibration to translate pixel coordinates and measured virtual depth to rectified coordinates in a 3D point cloud. The FOV that we metrically calibrated previously is 50×50×30mm [23]. This smaller FOV is chosen in the center of the image plane for increased accuracy.

After calibration and registration, we can transform coordinates from one camera to another as seen in Fig. 2b. We observe NIRF markers with the NIR camera and transform their locations into the 3D point cloud.

### C. NIRF Marker Design and Application

The biocompatible NIRF markers are created by mixing 2 mm^3^ of ICG (IC-GREEN, Akorn, Inc) with 0.5 milliliters Acetone and 0.25 milliliters (265 mg) cyanoacrylate (Permabond 102) for a consistency viscous enough to remain on tissue in an irrigated surgical field, yet fluid enough to permit easy application of the markers. Acetone is used as a solvent for the ICG and cyanoacrylate; it is mildly toxic and flammable. However, Acetone has been studied extensively and is recognized to have low acute and chronic toxicity, currently being used in a variety of consumer products from cosmetics to processed foods [29]. The viscosity of the cyanoacrylate used is 70-90 cP, and it hardens within 5-10 seconds, slowing down evaporation and direct absorption through the tissue. Even in the worst case, where 100% of the marker is absorbed immediately into the body, the systemic exposure to a human of average size (62 kg) would be less than 9 ppm per marker. No indications of toxicity in humans were reported after exposures to 2,100 ppm of Acetone for 8 hours/day [30]. Our application could be contrasted with the application of Aceton-based nail polish removers (30%-60% Acetone), which are applied more liberally, left to be absorbed for a longer amount of time, and applied chronically over one's life. A disposable syringe with 25 gauge needle is used to apply about 10 μL of the solution for each NIRF marker in a roughly circular shape on the surface of the tissue. The applied marker hardens and bonds to the tissue within seconds, and the tissue does not need to be prepared in any way to receive the marker. Markers adhere to irregular or wet surfaces. Once placed, the markers remain optically stable in the surgical field, are robust to tissue motion and occlusion, and can be left in the surgical site or gently peeled off with tweezers at the end of the procedure. ICG and cyanoacrylates have both been approved for clinical use by the FDA, albeit for other indications. ICG has been used intravenously for years at higher dosages (1-5 ml as opposed to 0.002 ml per marker in our experiments) [17], [18]. The particular type of cyanoacrylate used in these experiments, Dermabond, has been implanted in muscle for 90 days without adverse effects, shows no toxicity at dosages of 500mg/kg/day [31], and is often used as a medical adhesive for wound closure in greater quantities. Since the individual components of our marker are used medically and do not pose a serious risk, we believe that the composed marker exhibits good biocompatibility.

### D. Tracking Software

Tracking of descriptive 3D features within a surgical field is not an easy task. Organ surfaces are typically smooth and have similar visual appearances, and may also be occluded by blood or tissue. The motivation behind using NIRF markers is to create high contrast areas on the 2D NIR image, which can be reliably tracked and projected onto the 3D point cloud obtained by the plenoptic camera to track the 3D coordinates of the NIRF markers.

The gain and exposure parameters of the NIR camera are set to ensure healthy contrast between the tracked blobs and surrounding environment, and to accommodate changing lighting and scene conditions, or occlusion by tissue or blood. Suitable values for gain and exposure are found to be 40 (data number/electrons) and 50,000 μs respectively. These values will vary depending on the type of camera used. In our ex-vivo experiments, the gain and exposure were set while observing markers occluded by tissue, and held constant throughout the ex-vivo tests. This ensures that all ex-vivo results have relevance in dynamically occluding environments.

**Figure 3:**
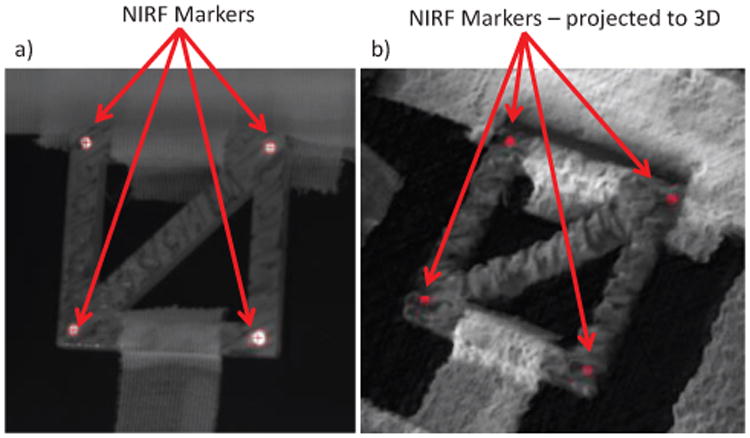
Showing a) NIR image with markers tracked by the ViSP library and their projection into co-registered 3D plenoptic camera point cloud in b). This projection follows the pipeline described in Fig. 2, and results in the 3D position of each tracked marker in metric coordinates.

The NIRF tracking is achieved by a custom C++ program written for ROS using the vpDot2 class of the Visual Servoing Platform (ViSP) library [32]. The trackers are initialized by their center of gravity in terms of pixel coordinates, which can be specified by clicking on the NIRF marker. Once initialized, the vpDot2 class returns the coordinates and higher order moments of a tracked blob of high-contrast intensity pixels (Fig 3a).

Tracking parameters include the maximum and minimum grey level intensity thresholds (set to 100 and 255), shape information (ellipsoid) and blob size precision (set to 0.1). The minimum intensity was determined to allow robust tracking while not confusing markers with surrounding tissues, which typically have intensity well under 100 at the proposed gain and exposure settings, while markers often have intensities of 165 or more. While tracking, estimations of marker position are informed by prior marker position, size and velocity. The tracking module computes the center of gravity of the tracked blob from the Freeman chain elements [33]. If the blob is lost (for example due to tool occlusion), a bounding box is searched around the previous position and tracking resumes once the marker reenters the box. The corresponding 3D point for each tracked marker is determined by projecting the point cloud onto the NIR image using the OpenCV function projectPoints [34], and taking the median of all pixels within a 5 pixel radius of the blob center of mass (Fig 3b), using knowledge of the NIR camera intrinsic parameters and the registration to the metrically calibrated 3D camera shown in Fig. 2.

2D image tracking is fast (∼19 Hz). However, calculating 3D metric coordinates from the plenoptic camera requires searching through the entire point cloud, which is slower than the plenoptic camera capture framerate of 5 Hz (Fig. 6). The hardware used to calculated depth from the raw plenoptic image is a Windows 7 computer with 4GB RAM, i7-3770 CPU running at 3.4 Ghz, and a GeForce GTX Titan graphics card with 2688 CUDA cores clocked at 836 MHz with 8GB of graphics memory. Our solution is to use the slower 3D information from the plenoptic camera to calculate metric XY coordinates at the faster rate of 2D tracking. One can use the depth information from the plenoptic camera, along with the NIR camera intrinsic parameters, to update the metric XY coordinates of a tracked marker at a faster rate as described in (4):

(4) $${\left[x,y\right]}_{n}=(\frac{{\left[u,\nu \right]}_{n}-{\left[C_{x},C_{y}\right]}_{n}}{{\left[f_{x},f_{y}\right]}_{n}})*(H_{n}^{p}*z_{p})$$

Where [*x*, *y*]*_n_* are the resulting *x* and *y* metric coordinates in the NIR camera frame, [*C_x_*, *C_y_*]*_n_* is the principal point in the NIR image, [*f_x_*, *f_y_*]*_n_* are the *x* and *y* focal lengths of the NIR camera, and [*u*, *ν*]*_n_* are the reported NIR image coordinates of the tracked blob. The z*_p_* value is the metric depth in the plenoptic camera frame, transformed into the NIR frame by $H_{n}^{p}$, calculated at registration. This metric depth is updated at the plenoptic framerate while [*u*, *ν* ]*_n_* are updated at the faster NIR camera framerate. Resulting metric *x* and *y* coordinates are calculated at the faster rate while *z* updates at the slower rate. This hybrid 2D/3D approach allows our system to track dynamic scenes faster and more accurately than either camera in isolation.

### E. Tool Tracking Test

**Figure 4:**
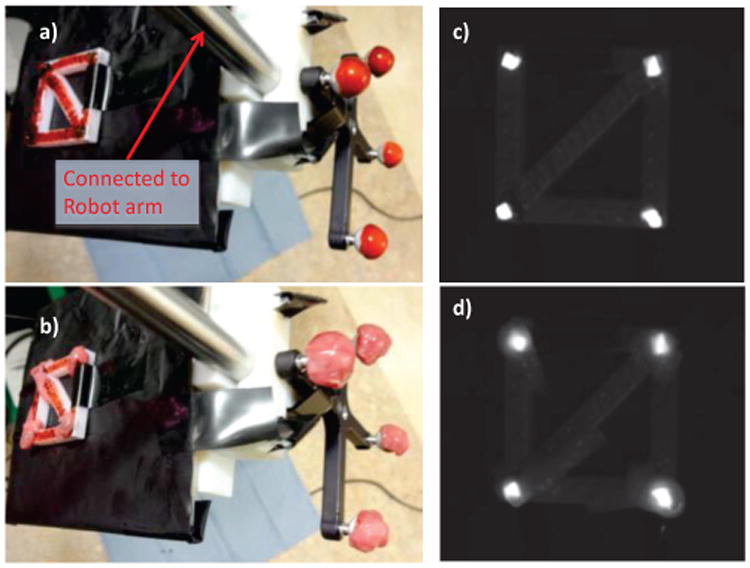
Tool tracking test during occlusion conditions. Both marker types obscured by a) blood and b) tissue, mounted to a robotic arm reference. The infrared image shows NIRF markers clearly visible while covered by c) blood and d) tissue. By comparing the observed trajectories from our NIR-3D tracker and a standard optical tracker to the robotic reference, we can evaluate tracking accuracy at various occlusion and speed conditions.

We tested the ability of our system to track scenes in motion with a mock “tool” (Fig 4), meaning a rigid fixture of four NIRF markers, and compared its performance to a commercially available optical tracker (Polaris Hybrid, Ontario, Canada) with framerate of 60 Hz and reported accuracy of 0.35 mm using a robotic reference (Light weight robot (LWR) 4+, KUKA Robotics Corporation, Augsburg, Germany) with a reported repeatability of 50 μm which was queried at a framerate of 100 Hz. Both optical (tool marker 8700338) and NIRF (n=4) markers were mounted to the robotic actuator as seen in Fig 4a, and positioned inside the NIR-3D system field-of-view along a predefined path. This path was a continuous motion along 4 diagonal lines which are maximally misaligned with our plenoptic camera frame, ensuring that we were tracking motion in all 3 XYZ coordinates simultaneously inside a 50×50×30 mm FOV. The tracking tests were conducted at 1, 5, and 10 mm/s to evaluate accuracy at speeds exemplary of typical tool and respiratory motion. While tracking, we recorded time-stamped position measurements from the robotic arm, the Polaris, and our NIR-3D system. The time-stamping occurred when the metric coordinate is calculated, not when the image is acquired. This allowed us to compare each measured point (the average of the four markers) against the reference point that is closest in time, which exposes error due to system lag. After an initial rough alignment manually, both observed point clouds from the NIR-3D and Polaris were aligned with the robotic tool path by an iterated closest points (ICP) method [35]. The ICP registration minimizes the error between the point clouds, and so will only reveal errors due to distortion of the expected trajectory, not errors due to imperfect registration of the robot and camera frames, or errors in manual alignment. If ICP was not used, errors in hand-eye registration would be present in the results, obscuring the true error of the NIR-3D vision system alone. Additionally, the ICP registration allows a fair comparison between the optical tracker and our 3D-NIR system. After ICP registration, error for all points was then calculated as the RMS distance from the measured point to the corresponding robotic reference point closest in time, to expose system lag. We broke down the NIR-3D system error into XYZ components in the NIR camera frame. We also calculated the spatial NIR-3D error i.e. the norm error between measured and reference points closest in space, which does not account for any error parallel to the robot trajectory. Additionally, the 95% one-sided confidence interval was computed, giving the range that the error would fall within 95% of the time.

The tracking test was repeated at 1 mm/s with both sets of markers covered in blood to simulate a surgical field. We used artificial blood (Rubie's Costume Company, Richmond Hill, NY) as seen in Fig. 4a. Artificial blood was used due to consistency, ease of storage and contamination concerns. The optical properties of real blood vary widely with physiological conditions such as osmolarity, flow conditions, and haematocrit [36], making simulated blood a more stable and repeatable choice. Simulated blood has been used for imaging experiments previously while investigating spectral interferences [37] and as a reference sample in optical imaging analysis [38]. The blood obscured the markers from view by the trackers, and is similar in appearance and consistency to real blood. In section IIG, *in-vivo* experiments detail the use of real blood with our NIR-3D system, which we have used previously under blood occlusion *in-vivo* [25].

We repeated the tracking test at 1 mm/s with markers covered in a single layer of porcine small bowel tissue (thickness ∼1.3 mm). This simulated tissue occlusion during surgery. As seen in Fig. 4b, the tissue completely covered the markers, making them difficult to see.

To investigate the speed of our tracking system with different numbers of trackers, we increased the number of tracked markers from 1 to 10 in the FOV and quantified the change in system speed for the full 3D calculation and the faster hybrid tracking. With each repetition, we added one marker to the scene and tracked with our system. Sufficient spacing between markers was required to ensure they were not confused with each other during tracking. For this purpose we spaced markers at least 5 mm apart. The minimum suggested spacing depends on the camera settings, target speed, and scene lighting, as well as the crispness of the NIRF marker edges.

We also tested the brightness of the markers over time. This was done to ensure that they could be reliably tracked over the course of a procedure without a change in brightness and possibly left in the body for long-term tracking applications. The average signal intensity of an NIRF marker, imaged daily using identical lighting conditions, was quantified over 7 days using the ViSP library. The NIRF marker was exposed to natural and artificial lighting in the lab during the seven day test period.

### F. Tissue Tracking Test

We tested our NIR-3D system performance when the markers are adhered to tissue, which is difficult to achieve with other marker types and enables us to perform tissue tracking during surgery. We fixed a layer of excised porcine bowel to the robot and dispensed 4 NIRF markers on top of the tissue, to be tracked by our 3D-NIR system along the same trajectory as the tool tracking test at 1 mm/s. This test was repeated with the occlusion conditions of the tool test, while markers were covered in artificial blood and with another layer of tissue. We also characterized the average single-marker error on tissue, in addition to the error of the averaged path of all four markers. This is so that the reader may compute expected error for various tissue deformation or registration models.

### G. In-Vivo Tests

Excised tissue may exhibit different optical characteristics than seen during surgery. For this reason we tested our systems ability to handle occlusion in-vivo. We placed 3 markers of each type (optical and NIRF) on the skin of a sedated, intubated porcine model underneath blood and exposed intestinal mesentery tissue in a study approved by our Institutional Animal Care and Use Committee (IACUC). This tested our systems ability to handle blood and tissue occlusion in a realistic clinical environment.

## III. Results

### A. Tool Tracking Test

Our system was able to track with no loss of markers, and achieved near-millimeter accuracy in the static case. At 1 mm/s, the error was 1.61 mm, with the 95% one-sided confidence interval at 1.27 mm. For the Polaris, these numbers are 0.29 and 0.18 mm. The spatial error for our system was 0.87 mm in the static case. We attribute the slight degradation in performance using the Polaris in the static case to drift errors, which are exacerbated by longer testing times for the static case.

When the scene was covered in blood, our system saw almost no drop in performance as seen in Table 1. The error for our system with blood occlusion was 1.62 mm. The Polaris system was unable to track the markers.

With both the optical and NIRF markers obstructed by a layer of 1.3 mm thick porcine bowel tissue, our system experienced only a slight degradation of tracking accuracy. The error rose to 1.71 mm. The Polaris was unable to track the obstructed markers.

**Figure 5:**
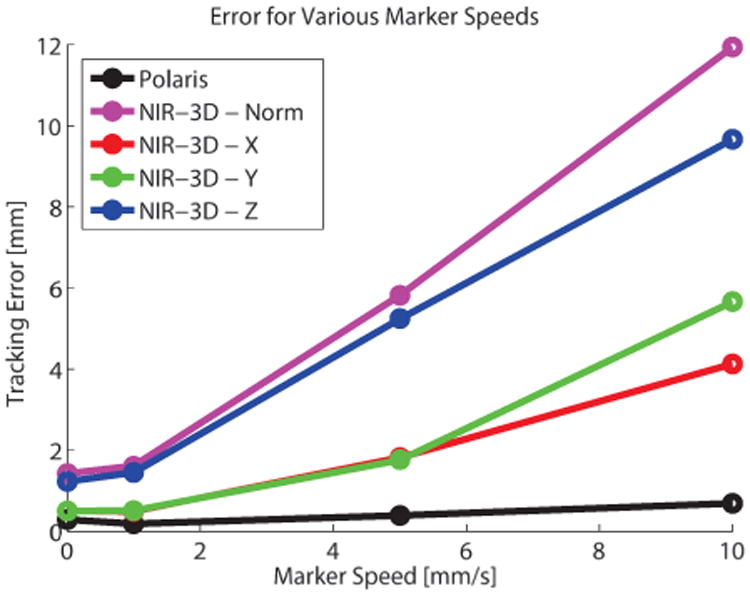
Visualization of tracking system error at increasing marker speed. Due to higher computation time, our NIR-3D system exhibits a lag, which causes error to increase linearly with marker speed. However, this error is dominated by the Z component, which is calculated from the plenoptic camera and updates more slowly than the XY components. Speeds tested were 0, 1, 5, and 10 mm/s.

The tool tracking test was repeated at speeds of 5 and 10 mm/s. Error increased for both trackers, particularly our NIR-3D system due to the relatively high system lag (Fig. 5). However, the XY error in the NIR camera frame was much lower than the Z error, due to the faster tracking in the XY plane and the plenoptic camera geometry, which triangulates depth from microlens images that are very close to one another.

**Figure 6:**
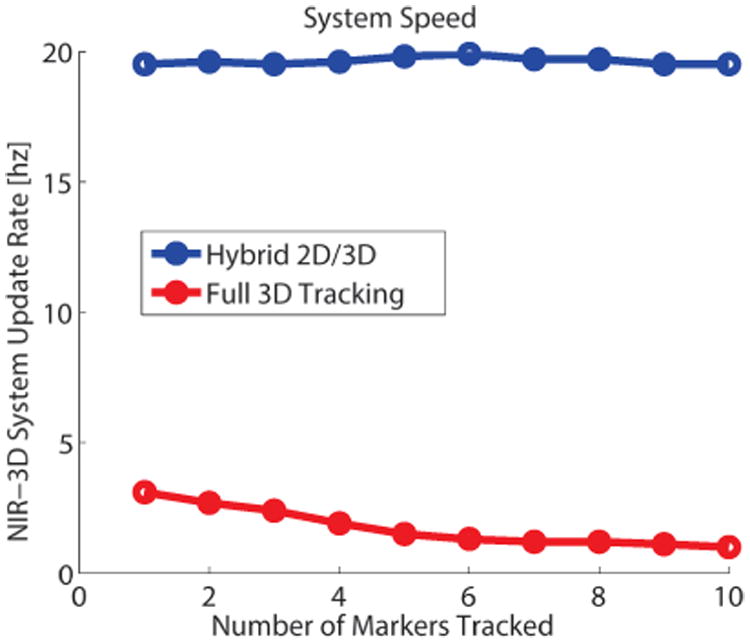
System throughput speed for full 3D tracking, where XYZ are all updated according to the plenoptic point cloud, and the 2D/3D hybrid approach described above, where the slower Z coordinate from the plenoptic camera is used with NIR camera parameters to update XY at a faster rate. While tracking more markers, the computational cost of tracking in full 3D increases, without affecting the hybrid method speed. This allows our system to track without losing high-speed markers.

Using only the NIR-3D system, we observed up to n=10 markers and measured the system framerate. The results showed that with more markers, the update rate of the system decreased due to an increase in computation time. This trend is visualized in Fig 6. However, by using intermittent depth information, the throughput markers tracked in 2D metric coordinates approaches the NIR camera framerate, regardless of the number of markers tracked.

**Figure 7:**
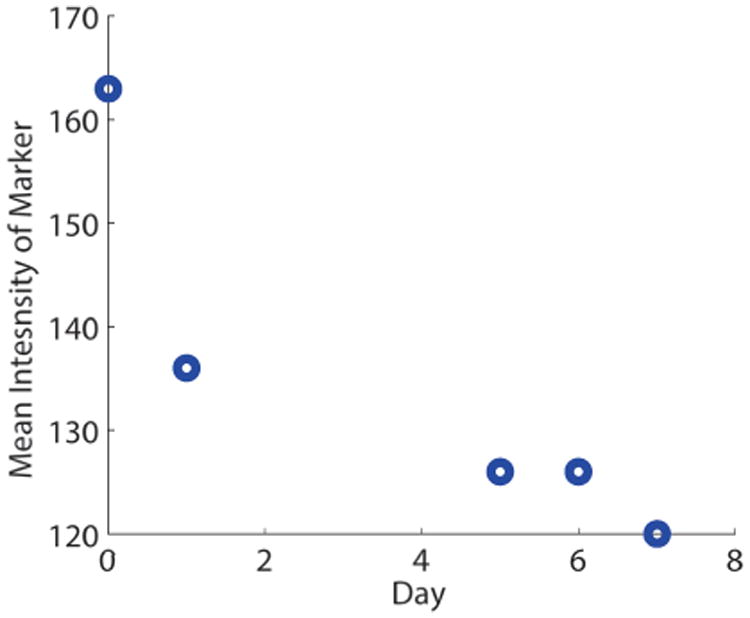
NIRF markers remain trackable for several days after creation, even without change to lighting conditions or camera parameters. Here the mean intensity, which can range from 0-255, stays above the minimum intensity threshold of 100 for 7 days.

Additionally, we monitored the average intensity level of a marker over a seven day period. The average signal intensity, meaning the average intensity of a tracked blob, decreased 26% in the seven days to a value of 120 (Fig 7). However, the markers can still be tracked as they are still well above the minimum threshold of 100. If the marker intensity decreases below the minimum threshold, then the system may be unable to track. Decreases in marker intensity do not affect system accuracy, however they may affect robustness to changes in illumination.

### B. Tissue Tracking Test

While observing markers on tissue, the norm 3D measurement error with our NIR-3D system was 1.83 (95% confidence interval of 1.49) mm. There was almost no difference between different occlusion conditions while tracking tissue as seen in Table 2. Single marker results are presented so that the reader may interpret expected error for various tissue modeling and tracking applications. The markers adhered firmly to tissue in all cases, and the viscosity allowed controlled application onto tissue.

### C. In-Vivo Test

**Figure 8:**
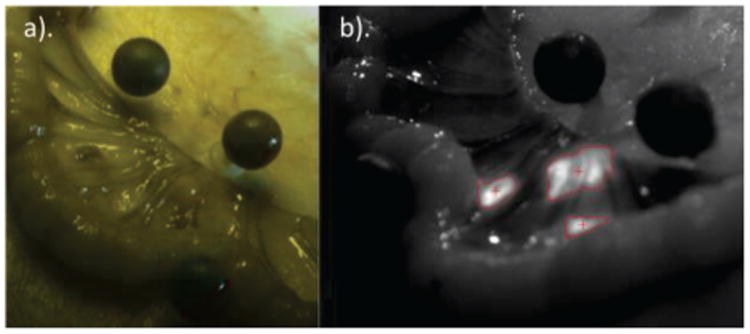
Near-Infrared Fiducial (nirf) Markers can be tracked in 3d while occluded by mesentery in-vivo. Part a) shows nirf markers occluded underneath tissue with surrounding optical markers in a color image. Part b) shows the nirf markers being tracked while underneath real tissue in an nir image. Even under occlusion and dynamic reflection, nirf markers can be tracked in-vivo.

Three markers of both types (optical and NIRF) were tracked in-vivo with no loss of markers in the unobstructed case. When splashed with porcine blood, the Polaris markers were unable to be seen. However, even when the NIRF markers were doused in blood and covered by a layer of bowel mesentery (Fig 8a), after adjustments to the NIR camera parameters they were tracked in the NIR image seen in Figure 8b. These adjustments were necessary because initial parameters were set in the unoccluded case.

## IV. Discussion

Our NIR-3D tracking system provided near-millimeter tracking accuracy at lower speeds, showing promise for the demanding clinical environment. However, the framerate of the full 3D tracking is currently too slow for more demanding tracking applications such as cardiac motion, where speeds can be upwards of 100 mm/s [3]. Additionally, tracking through tissue occlusion is only robust for thinner tissues (∼1.3 mm), which could be improved in future work, for example by using fluorescent dyes with higher signal yield and emission at a wavelength with deeper tissue penetration.

The general nature of our system permits the user to specify the number and location of markers to be tracked, encouraging tracking of amorphous surfaces typical in surgical environments. More importantly, the biocompatible NIRF marker enabled tracking even during occlusion by blood and tissue, which is not possible with current optical tracking methods.

The limiting factor in our tracking system is the framerate of the plenoptic depth calculation, which is currently 1.9 Hz while tracking 4 markers simultaneously. We mitigated this slow depth calculation with faster tracking in the NIR camera XY frame (19.6 Hz for 4 markers). Although the plenoptic camera has advantages compared to stereo cameras like a smaller footprint, the ability to provide a total-focus image, and imaging from multiple vantage points, the plenoptic depth calculation is more computationally expensive than stereo because of the higher number of microlens images, and the fact that every pixel is assigned a depth. However, with faster GPUs and more optimized correspondence searching, we believe these framerates can improve. In the tool tracking tests, we simply averaged the 4 marker trajectories to obtain a single tool trajectory, but we could use more advanced methods such as Kalman filtering [39] or a model of the 4 connected tool markers for enhanced accuracy.

This work has broader implications for real-time tracking of mobile deformable targets in unstructured surgical environments, even in situations where the line of sight to target of interest is obstructed. We specifically developed a novel biocompatible fluorescent dye-solvent to track target tissue, allowing real-time dynamic visualization. This unique fluorescent dye-solvent enables us to create custom fiducials to accurately follow deformable, mobile, wet tissue targets. This approach obviates the need for cumbersome EM tracking while overcoming the limitations of in-sight optical tracking. Furthermore, the fusion with plenoptic imaging technology permits accurate quantitative registration and knowledge of the target tissue in 3D, providing a real-time quantitative 3D topography of the surgical field, including the tissue of interest and surrounding surgical environment.

## V. Conclusion

We presented the design of key elements of our NIR-3D system including the biocompatible marker composition, camera registration and calibration, and software methods used. Our system was able to track markers that adhere to the tissue surface during blood and tissue occlusion. These markers remain luminescent for long periods of time. We saw accuracy errors of 1.42 mm in the best case, and only small changes in accuracy with varying occlusion conditions. Errors were dominated by the Z-component, making our hybrid 2D/3D approach useful to track at faster rates in the XY plane.

Future work will include further integration and evaluation of the tracking system in image-guided medical interventions such as robotic surgeries. We also propose to automate gain and camera parameter adjustment to maintain a consistent size of tracked marker through occlusion conditions. We will improve the accuracy and speed of the tracking system through improved metric calibration, automatic adjustments to camera parameters, and optimizations to allow faster runtimes.

## Figures and Tables

**Table I:** The tool test (markers on a rigid fixture as seen in Fig. 4) was repeated at various speeds and occlusion conditions. Occlusion tests are at 1 mm/s. The static test observed stationary markers along the same trajectory. Unoccluded trials were seen by both trackers, but the Polaris was unable to track markers covered in blood or tissue. Reported error for Polaris and NIR-3D system is distance to closest point in time, NIR-3D spatial error is distance to closest point in space. Values reported are the Root Mean Square Error (RMSE) and the 95% one-sided confidence interval (CI).

Tool Tests	Polaris Error: Norm RMSE (CI) [mm]	NIR-3D Error: Norm RMSE (CI) [mm]	NIR-3D X RMSE (CI) [mm]	NIR-3D Y RMSE (CI) [mm]	NIR-3D Z RMSE (CI) [mm]]	NIR-3D Spatial Error: Norm RMSE (CI) [mm]
Static Test	0.29 (0.24)	1.42 (1.09)	0.50 (0.38)	0.50 (0.36)	1.23 (0.96)	0.87 (0.89)
1 mm/s	0.18 (0.14)	1.61 (1.27)	0.49 (0.37)	0.51 (0.39)	1.45 (1.15)	0.78 (0.80)
5 mm/s	0.39 (0.33)	5.82 (4.94)	1.82 (1.34)	1.76 (1.19)	5.24 (4.60)	2.61 (2.71)
10 mm/s	0.69 (0.61)	11.94 (11.00)	4.13 (3.38)	5.66 (5.01)	9.67 (9.19)	5.69 (6.01)
Blood occlusion	n/a	1.62 (1.28)	0.49 (0.38)	0.51 (0.39)	1.46 (1.15)	0.79 (0.81)
Tissue occlusion	n/a	1.71 (1.36)	0.49 (0.39)	0.50 (0.38)	1.56 (1.24)	0.89 (0.91)

**Table II:** Tissue tracking results for the average trajectory of n=4 markers on tissue, with average single marker error for each of the 3 occlusion conditions. Tissue tracking tests were conducted at speeds of 1 mm/s. Reported RMSE is distance to closest point in time, spatial error is distance to closet point in space. Additionally, the 95% one-sided confidence interval is reported (CI).

Tissue Tests	NIR-3D Error: Norm RMSE (CI) [mm]	X RMSE (CI) [mm]	Y RMSE (CI) [mm]	Z RMSE (CI) [mm]	NIR-3D Spatial Error: Norm RMSE (CI) [mm]
Unoccluded	1.83 (1.49)	0.57 (0.44)	0.59 (0.46)	1.64 (1.35)	0.93 (0.95)
Single Marker Error	1.62 (1.65)	0.58 (0.59)	0.49 (0.49)	1.43 (1.45)	1.10 (1.12)
Blood occlusion	1.85 (1.52)	0.58 (0.45)	0.57 (0.45)	1.67 (1.38)	0.95 (0.97)
Single Marker Error	1.59 (1.62)	0.65 (0.67)	0.61 (0.63)	1.31 (1.34)	1.06 (1.09)
Tissue occlusion	1.83 (1.57)	0.65 (0.52)	0.67 (0.55)	1.57 (1.37)	0.96 (0.98)
Single Marker Error	1.08 (1.11)	0.60 (0.62)	0.52 (0.53)	0.73 (0.75)	1.08 (1.11)
